# Magnetoliposomes with Calcium-Doped Magnesium Ferrites Anchored in the Lipid Surface for Enhanced DOX Release

**DOI:** 10.3390/nano13182597

**Published:** 2023-09-20

**Authors:** Beatriz D. Cardoso, Diana E. M. Fernandes, Carlos O. Amorim, Vítor S. Amaral, Paulo J. G. Coutinho, Ana Rita O. Rodrigues, Elisabete M. S. Castanheira

**Affiliations:** 1 Physics Centre of Minho and Porto Universities (CF-UM-UP), Campus de Gualtar, 4710-057 Braga, Portugala92249@alunos.uminho.pt (D.E.M.F.);; 2 LaPMET—Laboratory of Physics for Materials and Emergent Technologies, Universidade do Minho, 4710-057 Braga, Portugal; 3 CMEMS—UMinho, Universidade do Minho, DEI, 4800-058 Guimarães, Portugal; 4 LABBELS—Associate Laboratory, 4800-058 Guimarães, Portugal; 5 Physics Department and CICECO, University of Aveiro, Campus de Santiago, 3810-193 Aveiro, Portugal

**Keywords:** stimuli-responsive magnetoliposomes, magnetic nanoparticles, cubic shape, doxorubicin, hyperthermia, controlled drug release

## Abstract

Nanotechnology has provided a new insight into cancer treatment by enabling the development of nanocarriers for the encapsulation, transport, and controlled release of antitumor drugs at the target site. Among these nanocarriers, magnetic nanosystems have gained prominence. This work presents the design, development, and characterization of magnetoliposomes (MLs), wherein superparamagnetic nanoparticles are coupled to the lipid surface. For this purpose, dimercaptosuccinic acid (DMSA)-functionalized Ca_0.25_Mg_0.75_Fe_2_O_4_ superparamagnetic nanoparticles were prepared for the first time. The magnetic nanoparticles demonstrated a cubic shape with an average size of 13.36 nm. Furthermore, their potential for photothermal hyperthermia was evaluated using 4 mg/mL, 2 mg/mL, and 1 mg/mL concentrations of NPs@DMSA, which demonstrated a maximum temperature variation of 20.4 °C, 11.4 °C, and 7.3 °C, respectively, during a 30 min NIR-laser irradiation. Subsequently, these nanoparticles were coupled to the lipid surface of DPPC/DSPC/CHEMS and DPPC/DSPC/CHEMS/DSPE-PEG-based MLs using a new synthesis methodology, exhibiting average sizes of 153 ± 8 nm and 136 ± 2 nm, respectively. Doxorubicin (DOX) was encapsulated with high efficiency, achieving 96% ± 2% encapsulation in non-PEGylated MLs and 98.0% ± 0.6% in stealth MLs. Finally, drug release assays of the DOX-loaded DPPC/DSPC/CHEMS MLs were performed under different conditions of temperature (37 °C and 42 °C) and pH (5.5 and 7.4), simulating physiological and therapeutic conditions. The results revealed a higher release rate at 42 °C and acidic pH. Release rates significantly increased when introducing the stimulus of laser-induced photothermal hyperthermia at 808 nm (1 W/cm^2^) for 5 min. After 48 h of testing, at pH 5.5, 67.5% ± 0.5% of DOX was released, while at pH 7.4, only a modest release of 27.0% ± 0.1% was achieved. The results demonstrate the potential of the MLs developed in this work to the controlled release of DOX under NIR-laser stimulation and acidic environments and to maintain a sustained and reduced release profile in physiological environments with pH 7.4.

## 1. Introduction

Cancer consists of a group of diseases (more than 100) characterized by the uncontrolled growth and spread of abnormal cells [1,2]. Despite significant advancements in the field, the current options for cancer treatment have remained largely unchanged over recent decades, encompassing surgery, radiotherapy, chemotherapy, and their combinations [1,3]. In turn, chemotherapy is indicated as the treatment modality with the most significant therapeutic effectiveness, being employed as a primary induction or adjuvant treatment [4,5]. Antineoplastic drugs—such as doxorubicin (DOX)—that target rapidly dividing cells are usually used in this type of therapy [6,7,8]. This mechanism of action is associated with a lack of selectivity of the agents, resulting in reduced bioavailability at the target site and, consequently, the unspecific distribution of the drug in the healthy tissues, causing severe side effects [9].

Anticancer drug delivery using lipid-based systems has been explored as a promising cancer treatment for improving drug bioavailability and selectivity [10,11]. In turn, liposomes are one of the best-investigated drug nanocarriers due to their structural and compositional versatility, biocompatibility, morphological similarity with cell membranes, and ability to incorporate hydrophilic compounds (aqueous core) and/or lipophilic compounds (in the bilayers) [12,13,14,15]. Furthermore, these lipid vesicles can enhance the selectivity of drug accumulation in solid tumors due to a passive targeting via enhanced permeability and retention (EPR) effect [16,17,18]. The EPR effect is promoted by the pathophysiological characteristics inherent to solid tumors, such as (i) irregular neovascularization and structural and functional abnormalities in tumor blood vessels [19,20,21] and (ii) the lack of efficient drainage of tumors’ lymphatic systems [16,22,23]. Considering that the EPR effect is highly dependent on the circulation time of liposomes in the bloodstream, modifications to the surface of liposomes have been proposed to enhance their circulation time [24]. The covalent linking of polyethylene glycol (PEG) chains to liposomes’ surface, known as PEGylation, is the most used approach to obtain sterically stabilized liposomes (stealth liposomes) [25,26,27,28].

Despite the advantages of these approaches, conventional liposomes have limitations in terms of controlling drug release in both time and space and exhibit constraints with drug–target interaction and drug-release efficacy at the target site [29,30]. Combining magnetic nanoparticles with liposomes, forming a supramolecular hybrid lipid structure called a magnetoliposome, is a promising proposal to address some limitations [31,32,33]. Magnetic nanoparticles, particularly superparamagnetic iron oxide nanoparticles (SPIONS), are especially appealing for cancer treatment because they can be manipulated with a high-gradient external magnetic field, allow magnetic resonance imaging, and have heat generation ability when subjected to external energy sources [34,35,36,37,38]. The most commonly used energy sources are alternating magnetic fields (AMF) [39,40,41,42]—magnetic hyperthermia—and near-infrared (NIR) laser light irradiation [43,44,45]—photothermal therapy. One of the advantages of using NIR-induced photothermal hyperthermia over AMF-induced hyperthermia is related to its higher tissue-penetrating capabilities, being minimally invasive and more targeted to a specific part of the body [46,47]. In turn, hyperthermia is an adjuvant therapy proposed for the treatment of cancer, where tumor cells are affected by the rise of the local temperature to values between 43 °C and 46 °C [47,48]. The increase in temperature enhances the chemotherapy therapeutic effect by inducing greater perfusion within the tumor, which leads to a larger internalization of chemotherapy drugs [49,50,51]. It also promotes an increase in blood circulation and, consequently, greater oxygenation, making tumors more sensitive to the action of radiotherapy [52,53,54,55].

Ca-doped magnesium nanoparticles (Ca_0.25_Mg_0.75_Fe_2_O_4_) are of particular interest because, in addition to their excellent biocompatibility and high magnetization, have been reported as hyperthermia agents [56]. Furthermore, due to their high near-infrared (NIR) absorption and strong PT conversion efficiency, these ferrite nanoparticles have demonstrated potential as PTT agents, making them promising for more efficient and safe heating treatment approaches [57]. The present work is devoted to developing MLs with DMSA-coated calcium-doped magnesium ferrite nanoparticles with shape anisotropy (NPs@DMSA) coupled to the lipidic surface and a novel method to produce them. It aims to elucidate the implications of this spatial distribution through a comparative analysis with previously investigated architectures and methodologies (namely solid and aqueous magnetoliposomes). By assessing its effects on critical parameters such as colloidal stability, nanoparticle encapsulation efficiency, drug encapsulation efficiency, and photothermal capability, this study provides insight into the advantages and limitations of this type of magnetoliposomes’ architecture. Furthermore, the effects of different lipid compositions on the variation of physicochemical, structural, and stability properties of MLs were studied to ensure that the synthesis of MLs follows the needs of biomedical applications. The main aim of studying several different lipid combinations is to obtain MLs responsive to multiple stimuli (thermal and/or pH sensitivity) and, with this, to increase the control of drug release under the acidic microenvironment of tumors and hyperthermia conditions.

## 2. Materials and Methods

All the synthesis procedures used ultrapure water Milli-Q grade (MilliporeSigma, St. Louis, MO, USA) and spectroscopic grade solvents.

pH buffer stock solutions in the pH range between 2 and 11 were prepared from a sodium phosphate (Na_3_PO_4_, from Sigma-Aldrich, St. Louis, MO, USA) 0.1 M solution, adjusting the pH by mixing properly a solution containing citric acid (HOC(COOH)(CH_2_COOH)_2_, from Sigma-Aldrich, St. Louis, MO, USA) at 0.05 M and boric acid (H_3_BO_3_, from Sigma-Aldrich, St. Louis, MO, USA) at 0.2 M in ultrapure water, following [58]. The pH values were measured with a pH meter NiCd-1 (ORION, SA250 PH Meter Digital), and each solution pH was correctly adjusted using HCl or NaOH solutions (1 M).

For ML preparation, the lipids dipalmitoylphosphatidylcholine (DPPC) (from Sigma-Aldrich, St. Louis, MO, USA), distearoylphosphatidylcholine (DSPC) (from Sigma-Aldrich, St. Louis, MO, USA), cholesteryl hemisuccinate (CHEMS) (from Sigma-Aldrich, St. Louis, MO, USA), and 1,2-distearoyl-*sn*-glycero-3-phosphoethanolamine-*N*-[methoxy(polyethylene glycol)-2000] (ammonium salt) (DSPE-PEG2000, from Avanti Polar Lipids, Birmingham, AL, USA) were used.

Fluorescence spectra were measured on a Fluorolog 3 spectrofluorimeter (HORIBA Jobin Yvon IBH Ltd., Glasgow, UK) equipped with Glan–Thompson polarizers and dual monochromators in excitation and emission. The fluorescence spectra were corrected for the instrumental response of the system.

Images of the magnetic nanoparticles were obtained by transmission electron microscopy, in a JEOL JEM1010 (100 kV) at the Center for Scientific and Technological Research Support (CACTI), of the University of Vigo, Spain. The images were processed using ImageJ software (version 1.53t, National Institutes of Health (NIH), Bethesda, MD, USA).

The electron microscopy images of the magnetoliposomes were obtained with a scanning electron microscope (SEM) model FEI Nova 200 NanoSEM, operating in transmission mode (STEM), from the Materials Characterization Services Laboratory at the University of Minho (SEMAT/UM).

### 2.1. Magnetic Nanoparticles Preparation

Shape-anisotropic Ca_0.25_Mg_0.75_Fe_2_O_4_ nanoparticles with shape anisotropy coated with DMSA were synthesized for the first time. The nanoparticles were initially synthesized with a previously established protocol [59,60]. In turn, the functionalization of the nanoparticles was carried out by replacing oleic acid with DMSA, following the methodology described by Roca et al. [61].

The procedure was started by heating 15 mL of octadecene to 120 °C under continuous magnetic stirring. Then, 160 mg of magnesium acetate tetrahydrate (0.75 mmol), 39.5 mg of calcium acetate hydrate (0.25 mmol), 526 mg of tribasic iron (III) citrate monohydrate (2 mmol), and 879 mg of oleic acid (3.1 mmol) were added to the octadecene solution. The solution was kept at 120 °C for 60 min, and after this period, the reflux condenser was connected. After that, the solution was heated to 200 °C with a heating rate of 5 °C/min, which was maintained at 200 °C for 10 min. Then it was heated to 290 °C at a heating rate of 1 °C/min and refluxed for 60 min. Subsequently, washings were carried out with ethanol through several cycles of centrifugation and magnetic decantation to obtain nanoparticles covered by oleic acid. Finally, to replace oleic acid with DMSA and thus obtain hydrophilic nanoparticles, a mixture of 50 mL of toluene and a solution of 45 mg of DMSA in 5 mL of DMSO (dimethyl sulfoxide) was added to the particles, which were sonicated for 5 min and mechanically shaken for 24 h. Subsequently, toluene was added to the reaction mixture, which was centrifuged again, and the supernatant, still containing particles coated with oleic acid, was discarded. Furthermore, multiple washing cycles with ethanol and acetone were carried out by centrifugation and magnetic decantation to remove the oleic acid molecules. Finally, the purified Ca_0.25_Mg_0.75_Fe_2_O_4_ nanoparticles coated with DMSA were dispersed in water and stored in a glass vial, at room temperature, for future use.

### 2.2. Structural Characterization of Magnetic Nanoparticles

X-ray diffraction (XRD) analyses were performed in a conventional Philips PW 1710 (Royal Philips, Amsterdam, The Netherlands) diffractometer, operating with CuK_α_ radiation, in a Bragg–Brentano configuration, from the University of Trás-os-Montes and Alto Douro.

Absorption spectra of magnetic nanoparticle dispersions were acquired using a Shimadzu UV-3101PC UV-Vis-NIR (Shimadzu Corporation, Kyoto, Japan) spectrophotometer with a 1 cm optical path length. The photothermal potential was evaluated, analyzing thermal energy dissipation under NIR radiation in a home-made setup. The experiment operation includes a sample holder connected to an adjustable temperature bath, a laser light source with a wavelength of 808 nm and 1 W/cm^2^ power density, and a T-type thermocouple connected to a digital multimeter Agilent U1242A (Agilent Technologies, Santa Clara, CA, USA) for temperature measurement. Before each measurement, the temperature was stabilized at body temperature (around 37 °C), and the samples were irradiated. The heating curve was recorded under laser irradiation for 30 min, and afterward, the laser was turned off, and the cooling temperature was measured for an additional 30 min. The heating ability was quantified by assessing the specific absorption rate (SAR) using the initial slope method by Equation (1) [62]:

(1) $$\mathrm{SAR}=C\frac{\Delta T}{\Delta t}\frac{m_{s}}{m_{m}}$$

in which C is the specific heat capacity of the aqueous medium (4.186 J·g^−1^·°C^−1^); ΔT/Δt is the initial slope of the heating curve as a function of time; and m_s_ and m_m_ the masses of the solvent and the magnetic material, respectively.

The magnetic characterization was performed at room temperature in a Superconducting Quantum Interference Device (SQUID) Magnetometer, MPMS3 SQUID (Quantum Design Inc., San Diego, CA, USA), at the Department of Physics and CICECO, University of Aveiro.

### 2.3. Preparation of (Magneto)liposomes with Surface-Coupled Magnetic Nanoparticles

DPPC-based liposomes were prepared by the ethanol injection method [63]. First, 1 mM of DPPC was dissolved in absolute ethanol. Then, the lipid ethanolic solution was added, drop by drop, to 3 mL of phosphate buffer solution (pH 7.4) pre-heated at 55 °C (above the transition temperature) under stirring in a vortex. To prepare DOX-loaded liposomes, 10 µM of DOX ethanolic solution was co-injected into the pre-heated buffer solution.

The synthesis of MLs with surface-coupled magnetic nanoparticle preparation followed the lipid compositions and the corresponding ratios presented in Table 1.

First, thin films of the lipid formulations (based on DPPC, DPPC/DSPC/CHEMS, and DPPC/DSPC/CHEMS/DSPE-PEG) at the corresponding ratios and a final concentration of 1 mM were prepared by solvent evaporation under an ultrapure nitrogen flow. After that, 3 mL of heptane (99%) was added to the thin film and ultrasonically processed (Misonix, Farmingdale, NY, USA, Touch-screen S-4000) at 190 W for 15 min to create reverse micelles of a uniform size. Then, the heptane was evaporated under an ultrapure nitrogen flow to obtain a thin film with inverted micelles. Next, 3 mL of a phosphate buffer solution (pH 7.4) was heated to 55 °C (above the lipid transition temperature) containing 2 mg of the prepared magnetic nanoparticles, which was then added to the lipid film. Finally, the mixture was sonicated at 60 °C for 1 h to produce MLs of suitable sizes.

The preparation of DOX-loaded MLs (based on DPPC, DPPC/DSPC/CHEMS, and DPPC/DSPC/CHEMS/DSPE-PEG) follows the methodology described above, in which 10 µM of an ethanolic DOX solution was added to the micelle film and followed by the addition of the phosphate buffer solution (pH 7.4) containing 2 mg of magnetic nanoparticles to the mixture.

The morphology of the MLs was visualized using a scanning electron microscope model NanoSEM FEI Nova 200 in transmission mode (STEM).

### 2.4. Dynamic Light Scattering Measurements

The average hydrodynamic diameter (D_H_), polydispersity index (PDI), MLs’ long-term colloidal stability, and zeta-potential (ζ-potential) dependence on the pH of the several MLs was investigated by dynamic light scattering (DLS). The DLS measurements were obtained using a Litesizer 500 DLS equipment, having three detection angles (15°, 90°, 175°) from Anton Paar (Anton Paar GmbH, Graz, Austria), using a diode laser of λ = 658 nm and 40 mW. Polystyrene cells were washed with ethanol and filtered deionized water before and after all the measurements.

For the D_H_ and PDI determination, liposomes and MLs were prepared as described in Section 2.3, and filtered five times through a 0.2 μm filter before all the measurements for removal of dust particles. In turn, the ζ-potential dependence on the pH value was determined by the resuspension of 500 μL of the previously prepared MLs or liposomes to 500 μL of the buffer stock solution at the corresponding pH (between 2 and 11). Next, the solutions were filtered five times through a 0.2 μm filter, and the final pH value was measured and adjusted adequately with HCl or NaOH solutions (at 1 M) using a mini-pH meter NiCd-1. The results are presented as mean and corresponding standard deviation from triplicate assays.

The long-term colloidal stability of MLs based on DPPC/DSPC/CHEMS and DPPC/DSPC/CHEMS/DSPC-PEG solutions was investigated. For that, the MLs (following the Section 2.3 procedure) were stored at 4 °C for 10 days, and the D_H_ and PDI of the samples were monitored during this period.

### 2.5. Quantification of DOX Encapsulation Efficiency

The quantification of the encapsulation efficiency of DOX (EE%) in liposomes and MLs was performed using fluorescence spectroscopy. The fluorescence emission spectra were measured on a Fluorolog 3 spectrofluorometer (HORIBA Jobin Yvon IBH Ltd., Glasgow, UK) equipped with double monochromators in excitation and emission. The prepared DOX-loaded lipid nanosystems (following the procedures described in Section 2.3) were placed in an Amicon^®^ Ultra-0.5 mL with filters with a pore size of 0.1 µm, centrifuged at 3000 rpm for 10 min, and the supernatant collected. The concentration of the non-encapsulated DOX (supernatant) was quantified by fluorescence spectroscopy, using λ_exc_ = 480 nm, slits of 4 nm, and the emission spectrum collected in the 490–750 nm range. Three independent measurements were performed for each system, and standard deviations (s.d.) were calculated. The drug concentration in the sample was calculated using a previously obtained calibration curve [59], and the EE(%) of DOX was calculated according to Equation (2).

(2) $$\mathrm{EE}\left(\%\right)=\frac{\mathrm{Initial}\;\mathrm{DOX}\;\mathrm{concentration}-\mathrm{DOX}\;\mathrm{concentration}\;\mathrm{in}\;\mathrm{the}\;\mathrm{supernatant}\;}{\mathrm{Initial}\;\mathrm{DOX}\;\mathrm{concentration}}\times 100$$

### 2.6. Quantification of Magnetic Nanoparticles’ Lipid Surface-Coupling Efficiency

The quantification of the Ca_0.25_Mg_0.75_Fe_2_O_4_ nanoparticles’ coupling efficiency to the lipid surface was determined by quantifying iron(III) chloride complexes in the samples. This assay was based on the addition of concentrated hydrochloric acid (HCl) to the magnetic NPs to promote the formation of iron(III) chloride complexes with a strong signal in the UV-Vis range [64]. First, the previously synthesized MLs (following the procedure described in Section 2.3) were dried under an ultrapure nitrogen flow until a lipid film was formed. Then, to promote the digestion of the NPs, 1.2 mL of hydrochloric acid (HCl, 37%) was added to the lipid film, sonicated for 5 min, and left to digest for 1 h. Next, the acid concentration was diluted to 4 M using 1.8 mL Milli-Q grade water. The absorption spectrum of the samples was then obtained. Finally, the calibration curve was obtained by digesting known masses of the same NPs following a linear fitting of at least seven points. Three independent measurements were performed for each sample, and standard deviations (s.d.) were calculated.

### 2.7. Interaction with Human Serum Albumin (HSA)

An aqueous solution of HSA with a fixed concentration of 0.2 mM (similar to the concentration of HSA in blood plasma) was titrated with DOX-loaded liposomes (DPPC), DOX-loaded MLs (DPPC, DPPC/DSPC/CHEMS, DPPC/DSPC/CHEMS/DSPE-PEG), and NPs@DMSA, following the same procedure described in a previous work [59]. For each assay, 1 µL of the sample was added between each increment, and the sample was allowed to stabilize at room temperature for 10 min. Then, the fluorescent emission of tryptophan residues was measured by fluorescence spectroscopy in each increment (λ_exc_ = 280 nm, integration time of 1 s, and the slit width set to 2 nm). Then, the changes in tryptophan residues’ maximum fluorescence emission intensity (λ_em_ = 344 nm) were calculated, and the results were expressed by plotting fluorescence quenching of HSA emission as a function of ligand concentration. The HSA quenching is described by Equation (3):

(3) $$\%\mathrm{quenching}=\frac{y_{\max}^{\;n}\;}{1+\frac{k_{d}}{\left[\mathrm{ligand}\right]}}\;$$

where $y_{\max}^{\;}\;$corresponds to the maximum fluorescence quenching registered, n is the number of binding sites, and $k_{d}$ is the dissociation constant. The affinity between protein and ligand is inversely proportional to $k_{d}$ and can be expressed as 1/$k_{d}$.

### 2.8. Doxorubicin Release Kinetics

The kinetics of DOX release from MLs was firstly quantified in different pH environments and temperature conditions to understand the effect of each stimulus in the controlled release profile of DOX. The assays were performed in phosphate buffer at pH 5.5 and pH 7.4 to simulate the drug release profile in the acidic tumor extracellular microenvironment and physiological fluids [65,66], respectively, at 37 °C and 42 °C, representing the physiological temperature and simulating the hyperthermia treatments [67]. A high-efficiency reusable 96-well Micro Dialysis Device, HTD 96b from HTDialysis, LLC (Wales Ferry, CT, USA), with regenerated cellulose dialysis membranes, was used. The assays followed the procedure described in [59,60]. The DOX release from MLs was quantified by measuring DOX (collected from the acceptor compartments at different time points) fluorescence at λ_em_ =590 nm (λ_exc_ =480 nm). The experimental DOX release profiles were fitted to different kinetics models (Weibull) using Prism 8 software (GraphPad Software, La Jolla, CA, USA).

For DOX release under NIR-laser irradiation, this methodology was adapted, in which each sample was irradiated by a laser at 808 nm (1 W/cm^2^) for 5 min after each aliquot. Then, the fluorescence intensity was measured (λ_exc_ = 480 nm) and the corresponding DOX concentration at each time point was measured. These assays were performed in quadruplicate.

## 3. Results and Discussion

### 3.1. Characterization of Magnetic Nanoparticles

#### 3.1.1. X-ray Diffraction and TEM Analysis

**Figure 1:**
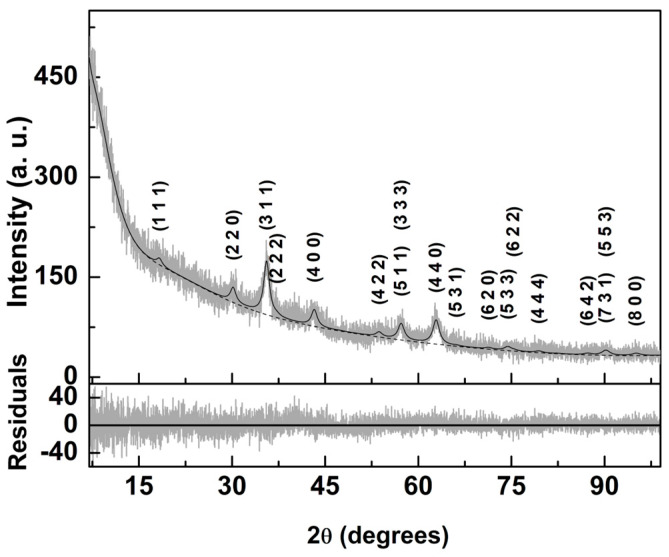
X-ray diffractogram of Ca_0.25_Mg_0.75_Fe_2_O_4_ nanoparticles functionalized with dimercaptosuccinic acid and fitting using a Rietveld analysis. Miller indices are indicated.

In this work, Ca_0.25_Mg_0.75_Fe_2_O_4_ nanoparticles coated with DMSA were synthesized. X-ray diffraction analysis gives information on the crystallinity and purity of the obtained magnetic NPs. The XRD diffractogram shown in Figure 1 was interpreted using Profex software (version 4.3.6) [68] which implements BGMN Rietveld [69]. Upon importation and adaptation of CIF file number 1,011,245 (space group Fd-3m:1) for MgFe_2_O_4_, it was necessary to change the unit cell composition, in order to be in accordance with the expected synthesized mixed ferrite; the position occupied by magnesium ions was considered to be 25% calcium and 75% magnesium. Further, a varying degree of inversion was taken into account, considering that the distribution of those two cations between tetrahedral and octahedral occurs so that the mixed ferrite stoichiometry is valid in both type of sites. All the peaks in the XRD diffractogram were accounted for, which proves the crystallographic purity of the prepared sample. The Ca_0.25_Mg_0.75_Fe_2_O_4_ diffraction peaks appeared at 18.4° (1 1 1), 30.2° (2 2 0), 35.6° (3 1 1), 37.2° (2 2 2), 43.3° (4 0 0), 47.4° (3 3 1), 53.7° (4 2 2), 57.2° (5 1 1), 57.2° (3 3 3), 62.9° (4 4 0), 66.1° (5 3 1), 71.3° (6 2 0), 74.4° (5 3 3), 75.4° (6 2 2), 79.4° (4 4 4), 82.4° (7 1 1), 82.4° (5 5 1), 87.2° (6 4 2), 90.2° (7 3 1), 90.2° (5 5 3), 95.1° (8 0 0), and 98.0° (7 3 3). The corresponding fit was obtained with χ^2^ = 1.16, GoF = 1.08, and R_P_ = 8.3, with an inversion degree of 0.67 ± 0.06, which was lower than the value obtained for magnesioferrite in a previous study [70]. The obtained lattice parameter was 8.354 Å, which is slightly lower than the value reported in the magnesioferrite CIF file (8.360 Å). The relative intensity and shape of the various diffraction peaks could be modeled using BGMN [69], considering only size-broadening effects and Debye–Waller factors (TDS). Using the full XRD pattern and the size-broadening effects implemented on BGMN [69], a crystallite size of 7.3 nm was obtained. The specific characteristics of the used XRD equipment were taken into account for calculating the instrument peak broadening through Profex software (version 4.3.6) [68].

**Figure 2:**
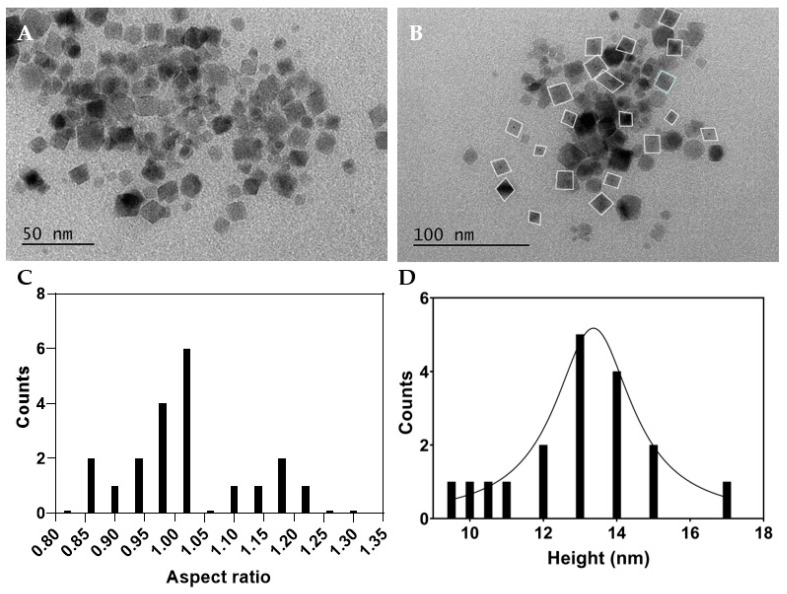
(**A**,**B**) TEM images of Ca_0.25_Mg_0.75_Fe_2_O_4_ ferrite nanoparticles functionalized with dimercaptosuccinic acid at different magnifications. (**C**) Aspect ratio distribution histogram of image B, and (**D**) size histogram of image B and fitting to a Lorentzian distribution (R^2^ = 0.95).

To assess the shape and size distribution of the Ca_0.25_Mg_0.75_Fe_2_O_4_ nanoparticles functionalized with dimercaptosuccinic acid, an electron microscopy characterization was performed. TEM images obtained are displayed in Figure 2.

The magnetic nanoparticles are shown to have a cubic shape, with an aspect ratio (height/width) between 0.85 and 1.3. These values were obtained from the measured mean length of the major and minor axes of the particles, followed by calculation of the corresponding aspect ratio (height/width). For the analysis of size distribution, the height values were considered and fitted to a Lorentzian distribution, centered at 13.36 nm with a width of 1.28 nm, comparing well with the crystallite sizes obtained by XRD.

#### 3.1.2. Photothermal Capability

**Figure 3:**
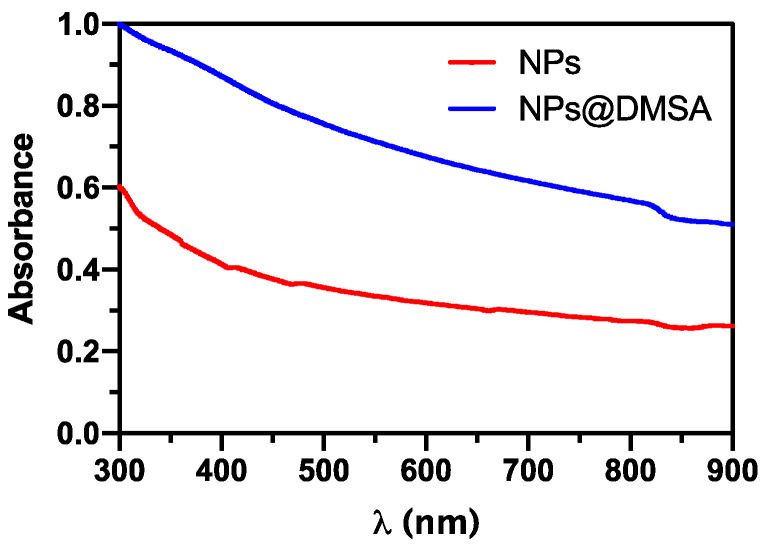
Absorption spectra of aqueous dispersions of non-functionalized NPs and of NPs@DMSA (2 mg/mL).

Figure 3 displays the UV-Visible-NIR absorption spectra of the aqueous dispersions of Ca_0.25_Mg_0.75_Fe_2_O_4_ nanoparticles (NPs) both before and after functionalization with DMSA. Both spectra demonstrate a large absorption and light scattering at shorter wavelengths, which are characteristic features of this type of ferrite nanoparticles [59,71]. An increase in absorption was observed for NPs@DMSA, due to larger light scattering from covered nanoparticles. These variations confirm the binding of DMSA to the surface of the magnetic nanoparticles.

**Figure 4:**
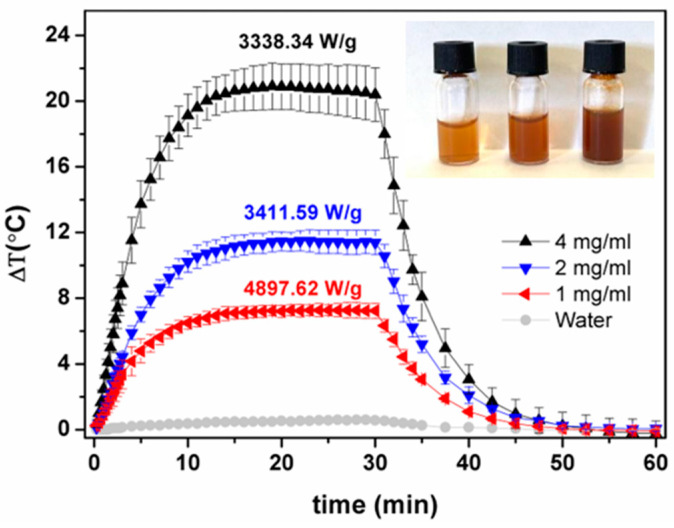
Heating and cooling profile of aqueous solutions of mixed ferrite NPs@DMSA, at different concentrations, under NIR light source with 808 nm wavelength and 1 W/cm^2^ power density. Inset: photographs of aqueous dispersions at different concentrations (from left to right: 1 mg/mL, 2 mg/mL, and 4 mg/mL).

The photothermal potential of iron oxide magnetic nanoparticles has been recently demonstrated [72]. In these assays, laser irradiation at 808 nm is widely used due to the first near-infrared (NIR-I) biological window (700–900 nm). For assessment of the photothermal hyperthermia potential of DMSA-functionalized magnetic nanoparticles (NPs@DMSA) under near-infrared light, aqueous dispersions of NPs@DMSA at various concentrations (1 mg/mL, 2 mg/mL, and 4 mg/mL) were examined, with the heating and cooling curves of water measured as a benchmark (Figure 4). The NPs@DMSA concentrations of 4 mg/mL, 2 mg/mL, and 1 mg/mL demonstrated maximum temperature variations of 20.4 °C, 11.4 °C, and 7.3 °C, respectively, during a 30 min irradiation period. Subsequently, upon laser interruption, all samples returned to their initial temperatures within approximately 20 min. The heating effect increased with higher particle concentration; however, the specific absorption rate (SAR) values displayed an inverse trend. This can be due to enhanced nanoparticles’ aggregation and particle–particle interactions at higher concentrations, compromising the dispersion stability, resulting also in larger measurement uncertainty (Figure 4).

Espinosa et al. [73] prepared iron oxide nanocubes with SAR values of 1200 W/g_Fe_ when subjected to laser irradiation with 808 nm wavelength and power density of 1 W/cm^2^ [73]. Thus, the higher SAR value obtained for the prepared mixed ferrite NPs@DMSA in the same irradiation conditions corroborates the potential of the prepared nanoparticles as PTT agents.

#### 3.1.3. Magnetic Properties

Magnetization measurements of nanoparticles, both before and after DMSA functionalization, were conducted at 300 K by SQUID. Corrections were made to the results for the geometric effects, and additional measurements were taken to correct the remaining field of the superconducting coil.

**Figure 5:**
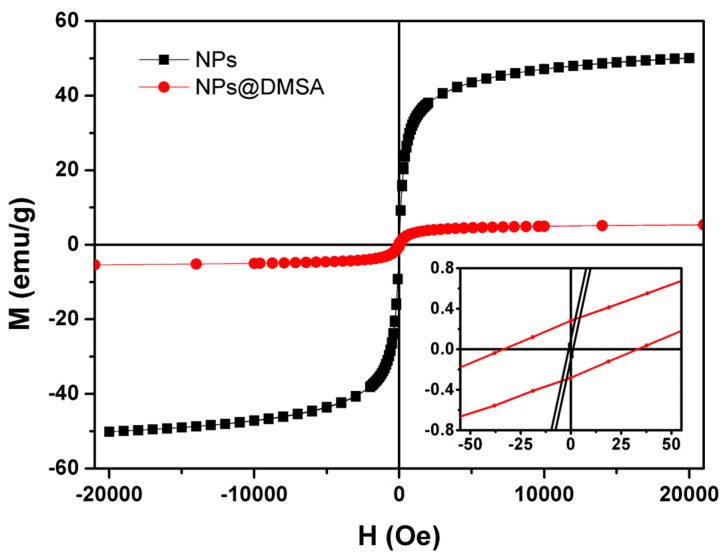
Magnetization hysteresis cycle of non-functionalized (black curve) and DMSA functionalized (red curve) nanoparticles of Ca_0.25_Mg_0.75_Fe_2_O_4_ at 300 K. Inset: magnification of the hysteresis loop in the low field region.

The magnetization curves, shown in Figure 5, exhibit a noticeable distinction between the nanoparticles that were not functionalized with DMSA (black curve) and those that underwent DMSA functionalization (red curve), when considering the saturation magnetization and coercive fields of the samples, as summarized in Table 2. The sample without DMSA functionalization displays a saturation magnetization approximately 9 times higher than that of NPs@DMSA. Furthermore, the NPs’ coercive field approaches the minimum detectable value of the equipment and is smaller than the estimated error range (between 0 and 2 Oe), indicating a significantly low value. The magnetic property differences between NPs and NPs@DMSA represent the successful functionalization of magnetic NPs with an organic layer of DMSA, whose diamagnetic contribution results in the obtained behavior.

The obtained hysteresis loop, characterized by its nearly closed shape, along with the reduced coercivity values, is consistent with the superparamagnetic behavior exhibited by the magnetic nanoparticles. According to Jung et al. [74], the remanence to saturation magnetization ratio (M_r_/M*_s_*) serves as a measure of the squareness of the hysteresis loops, where values below 0.1 indicate that 90% of the magnetization is lost after the removal of the magnetic field. Table 2 shows values significantly lower than this limit, proving that the nanoparticles synthesized here show superparamagnetic behavior. This behavior is associated with reduced remaining magnetizations, which benefits this type of application since it reduces the possibility of aggregation in vivo, representing an advantage over other types of nanoparticles [75].

### 3.2. Characterization of Magnetoliposomes

#### 3.2.1. Validation of Magnetoliposomes Synthesis with Surface-Coupled Magnetic Nanoparticles

This work focuses on the development and investigation of a novel magnetoliposome variant in which NPs@DMSA are coupled to the lipid surface, rather than occupying a solid inner core [59,60]. The main aim is to improve the drug-loading capacity of MLs by making the aqueous compartment available for DOX loading, while preserving (even increasing) the mass of magnetic particles per lipid vesicle [59]. To our knowledge, the ML preparation method scrutinized herein was described for the first time.

**Figure 6:**
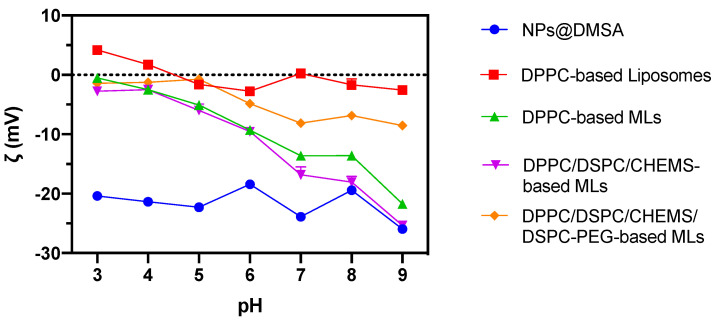
ζ-potential-pH profiles of NPs@DMSA, DPPC-based liposomes, and MLs based on several lipid compositions (DPPC, DPPC/DSPC/CHEMS, and DPPC/DSPC/CHEMS/DSPE-PEG) in pH range 2 to 11.

The initial stage of magnetoliposome development focused on monitoring the coupling of magnetic nanoparticles around the lipid bilayer through DLS measurements. For that, the ζ-potential-pH profiles (pH ranging from 2 to 11) of DPPC liposomes (used as a reference), NPs@DMSA, and MLs composed of three different lipid compositions were investigated (Figure 6). Liposomes (without magnetic nanoparticles) based on the zwitterionic lipid DPPC were found to have a near-neutral zeta-potential in all the pH ranges. On the other hand, the magnetic nanoparticles always showed a ζ-potential profile having negative values, varying only slightly between −20 mV (pH = 3) and −25 mV (pH = 9). The same type of assay was performed for MLs based on DPPC, DPPC/DSPC/CHEMS, and DPPC/DSPC/CHEMS/DSPE-PEG, the latter two being the lipid formulations of interest as a result of a pre-selection from previous work [60]. Similar to liposomes, DPPC-based MLs were investigated, since DPPC is the primary lipid of the MLs composition.

The MLs exhibited a distinct ζ-potential-pH profile, showcasing a hybrid system characterized by a combination of features from both liposomes and magnetic nanoparticles. In comparison to liposomes, MLs displayed a negative surface charge, indicating successful coupling of negatively charged nanoparticles onto the lipid vesicles’ surface. Notably, the negative surface charge was more prominent in DPPC and DPPC/DSPC/CHEMS-based MLs, than in stealth MLs formulated with DPPC/DSPC/CHEMS/DSPE-PEG. This result may arise from a reduced number of magnetic nanoparticles coupled in the latter system due to potential competition between DSPE-PEG molecules and the binding of magnetic nanoparticles to the lipid bilayer.

**Figure 7:**
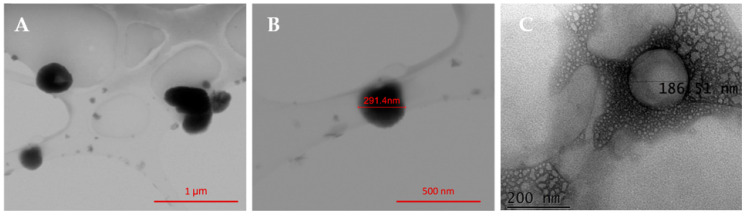
(**A**) SEM image of several DPPC/DSPC/CHEMS-based MLs with surface-coupled NPs@DMSA and (**B**) a single magnetoliposome. (**C**) TEM image of a neat liposome of DPPC/DSPC/CHEMS.

Following the pre-validation, the morphology of DPPC/DSPC/CHEMS-based MLs was observed by SEM. Figure 7A exhibits several high contrasting round-shaped MLs with an irregular surface that indicates nanoparticles’ coupling, while in Figure 7B a single magnetoliposome is represented. Moreover, the displayed MLs present an average size of 289 ± 25 nm. These results serve as a preliminary validation, confirming the suitability of the newly developed method for synthesizing MLs with NPs@DMSA coupled to the lipid surface. A TEM image of neat liposomes is shown in Figure 7C.

#### 3.2.2. Effect of Lipid Formulation on Structural and Colloidal Parameters of DOX-Loaded Magnetoliposomes

Understanding the impact of lipid composition on the structural and colloidal parameters of DOX-loaded magnetoliposomes is crucial to determine if the developed nanosystems meet the requirements for effective drug delivery. The lipid composition directly influences the lipid vesicles’ stability, electrical charge, rigidity, and size [76,77]. In turn, the hydrodynamic diameter (D_H_) of MLs and the corresponding polydispersity index (PDI) are primary factors in the initial characterization of lipid-based nanosystems. These factors control drug encapsulation efficiency, circulation half-life time, tissue diffusion, kidney excretion, and passive accumulation in the desired sites [78,79]. It is generally accepted that the size of these systems must vary between 50 and 200 nm [80] and that the PDI values must be equal to or less than 0.3, indicating a homogeneous population of the systems.

Similarly to the previous section, DPPC-based liposomes and MLs were characterized as a reference to assess the effects of incorporating DSPC/CHEMS and DSPC/CHEMS/DSPE-PEG into the lipid base. The obtained results are summarized in Table 3. The size of the DPPC-based magnetoliposomes (166 ± 3 nm) was found to be larger than that of the DPPC-based liposome counterparts (118 ± 5 nm), indicating the successful coupling of nanoparticles to the lipid vesicles and their impact on the system size. Additionally, the MLs based on the lipid formulations of interest (DPPC/DSPC/CHEMS and DPPC/DSPC/CHEMS/DSPE-PEG) exhibited sizes of 153 ± 8 nm and 136 ± 2 nm, respectively, which are suitable for various biomedical applications. The size reduction observed in PEGylated lipid formulations is typically attributed to the heightened lateral repulsion generated by PEG molecules [81]. This heightened repulsion leads to increased curvature in the lipid bilayer, ultimately decreasing vesicle size [81]. These MLs also demonstrated adequate PDI values, further confirming their homogeneous nature.

EE_DOX_ was calculated as the percentage of DOX encapsulated into MLs compared to the initially added concentration. The results are also summarized in Table 3. High EE_DO_ values were found in all systems, meeting the required criteria. In addition, one of the objectives of this investigation concerned the improvement of the EE_DOX_ in the MLs compared to the previously developed synthesis method [59]. The DPPC/DSPC/CHEMS-based magnetoliposomes showed a 4% increase in EE_DOX_, while the DPPC/DSPC/CHEMS/DSPE-PEG-based magnetoliposomes demonstrated a 6% increase in DOX encapsulation compared to the previous systems [60]. These improvements and the high EE_DOX_ evidence the suitability of the herein presented new method for the encapsulation of DOX and that the variation in lipid composition does not significantly impact DOX encapsulation.

The amount of NPs@DMSA coupled to the lipid surface (EE_NPs@DMSA_) is another crucial factor in the practical application of MLs. Therefore, the binding efficiency of NPs@DMSA to the MLs based on the lipid compositions of interest was determined by spectrophotometric determination of iron(III), and the results are summarized in Table 3. DPPC/DSPC/CHEMS-based MLs exhibited a higher EE_NPs@DMSA_ of (61 ± 3)% (equivalent to 0.52 mg of NPs@DMSA per 1 mM of total lipid). In contrast, the stealth MLs showed a lower EE_NPs@DMSA_ of 17% (equivalent to 0.15 mg of NPs@DMSA per 1 mM total lipid). These findings indicate a reduced ability of the NPs@DMSA to bind to the stealth MLs, possibly due to the competition between the DSPE-PEG molecules and the binding of the NPs@DMSA to the lipid bilayer. This behavior is consistent with the results obtained in Section 3.2.1, where the DPPC/DSPC/CHEMS-based MLs exhibited a more negative surface charge compared to the DPPC/DSPC/CHEMS/DSPE-PEG-based MLs. Additionally, the smaller hydrodynamic diameter observed in the latter can also be attributed to a lower number of NPs@DMSA present on the surface of the MLs.

#### 3.2.3. Interaction with Human Serum Albumin

The binding of proteins to drugs or nanocarriers plays a crucial role in the pharmacokinetics of a drug and, thus, is a key factor when assessing its therapeutic efficacy [82,83]. HSA is the most abundant protein in blood plasma and is known for its prominent role in drug binding [84,85]. HSA exhibits intrinsic fluorescence originating from tryptophan (Trp), tyrosine, and phenylalanine residues [86]. Among these residues, Trp214, located in the hydrophobic cavity of HSA, is responsible for its strong fluorescence emission [86]. Therefore, the interaction and binding of small molecules to HSA induce protein conformation changes, resulting in a fluorescence quenching of Trp fluorescence emission. Consequently, it allows the study of the drug/protein interaction by fluorescence spectroscopy [87,88,89]. Thus, more significant fluorescence quenching is associated with greater interaction and, as such, a reduced drug bioavailability.

**Figure 8:**
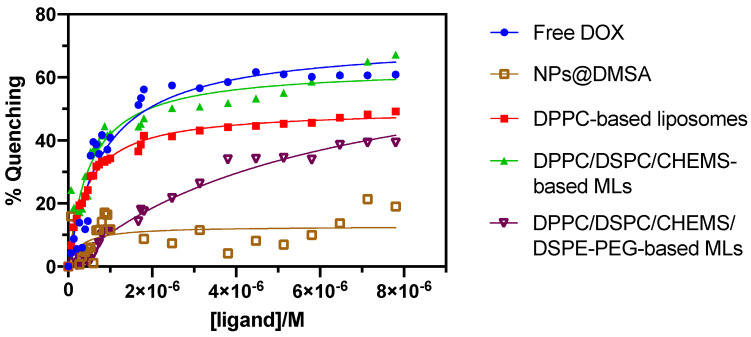
Quenching of HSA fluorescence (%) as a function of increasing concentration of titrated solutions with a non-linear fit, according to Equation (3).

The results of the interaction between free DOX, NPs@DMSA, and DOX-loaded MLs based on different lipid compositions (DPPC, DPPC/DSPC/CHEMS, and DPPC/DSPC/CHEMS/DSPE-PEG) are summarized in Figure 8. As anticipated, the titration of HSA with each corresponding treatment condition resulted in gradual fluorescence quenching as the concentration increased. However, the extent of quenching varied considerably between the different conditions.

To assess the contribution of NPs@DMSA to HSA quenching, the interaction of the latter with HSA was evaluated. A slight quenching effect was observed, although the behavior appeared to be irregular. Notably, a more pronounced increase in quenching was observed from a concentration of 5 × 10^−6^ M (indicated by a vertical dashed line in the Figure 8). The same behavior was verified for DPPC/DSPC/CHEMS-based MLs and DPPC/DSPC/CHEMS/DSPE-PEG-based MLs. Furthermore, DPPC/DSPC/CHEMS-based MLs demonstrated a higher quenching effect than previously developed DPPC/DSPC/CHEMS-based SMLs [60], whose maximum quenching values did not exceed 50%. Moreover, a typical behavior was found on DPPC-based liposomes. These results indicate that magnetic nanoparticles are coupled on the surface of liposomes, resulting in more erratic behavior. As previously described [60], PEGylated MLs have been shown to effectively reduce the interaction rate of MLs with HSA, playing an active role in the adequate protection of DOX that may enhance its bioavailability at the target site.

The obtained results were fitted according to a non-linear regression given by Equation (3), allowing the calculation of the dissociation constant ($k_{d}$), the binding constant ($k_{b}$), and the number of specific binding sites (n). The results are summarized in Table 4.

DPPC/DSPC/CHEMS-based MLs exhibited a higher binding constant and specific binding sites than DPPC-based liposomes. These findings can be attributed, in part, to the presence of magnetic nanoparticles on the lipid surface of MLs, which alone contribute to the binding constant ($k_{b}=3.58\times 10^{6}$ M^−1^). Additionally, the inclusion of DSPC in the lipid composition of MLs may further contribute to the enhanced binding properties observed. It is reported that DSPC liposomes bind more to serum proteins than DPPC liposomes [90]. In turn, the DPPC/DSPC/CHEMS/DSPE-PEG-based MLs present a $k_{b}=1.70\times 10^{5}$ M^−1^, substantially lower than all other conditions, namely free DOX and non-PEGylated counterparts. In the same way, the PEGylated formulation contributes to the reduction in specific binding sites to HSA, which is fundamental to providing cargo protection under physiological conditions.

#### 3.2.4. Stability of Magnetoliposomes upon Storage at 4 °C

**Figure 9:**
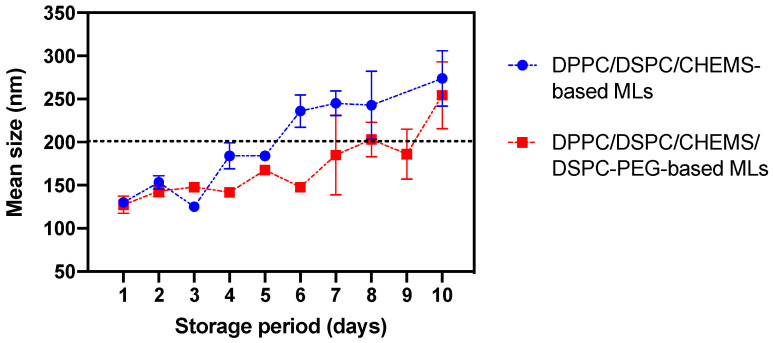
Stability of MLs based on DPPC/DSPC/CHEMS (non-stealth) and DPPC/DSPC/CHEMS/DSPC-PEG (stealth) expressed as variation in the mean size of formulations over a period of 10 days.

Evaluating the stability of liposomes during storage at 4 °C is of utmost importance as its shelf-life is crucial for the commercial and clinical use of the formulations, and because it directly affects the drug release kinetics and efficacy. A stability assay was conducted to evaluate the size variations of DPPC/DSPC/CHEMs and DPPC/DSPC/CHEMs/DSPE-PEG-based MLs over a period of 10 days. Nanosystems were stored at 4 °C, the most commonly used storage method for lipid vesicles in aqueous media. The findings are summarized in Figure 9. A slight trend of increasing average size was observed for the MLs over the days. The upper limit of 200 nm was chosen as a stability criterion for liposomal systems, as it is generally accepted that liposomes size should fall within 50 to 200 nm to be considered stable [91,92]. In this size range, nanosystems can avoid rapid clearance by the mononuclear phagocytic system (MPS), rapid uptake by the liver and spleen, and consequent elimination from the circulation and reduced aggregation and fusion [93,94,95,96]. In particular, in the non-stealth MLs the size variation become more pronounced from day 6 onwards, while keeping reasonable sizes below 300 nm. In turn, the stealth MLs exhibited a generally higher storage stability, approaching the size of non-PEGylated MLs at day 10.

#### 3.2.5. Release Kinetics of Doxorubicin under Physiological and Therapeutic Conditions

The DPPC/DSPC/CHEMS-based MLs were selected as optimal nanosystems for the proposed application, as they best respond to the physicochemical requirements for the application, displaying also the ability to encapsulate a greater amount of NPs@DMSA on the surface. As a result, they were subjected to further analysis to explore their capabilities for DOX release in detail.

**Figure 10:**
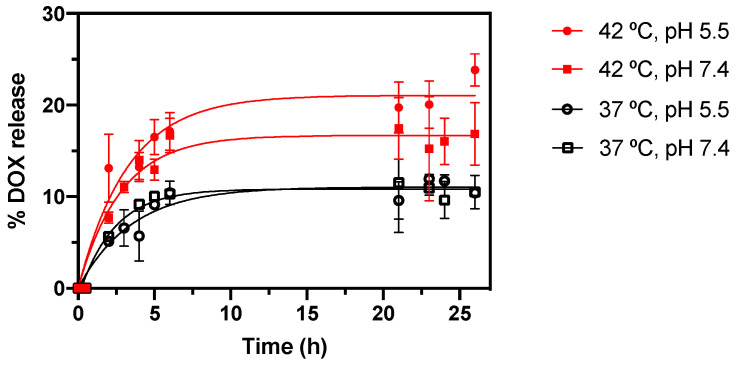
Profile of the in vitro release kinetics of DOX from DPPC/DSPC/CHEMS-based MLs under different temperature and pH conditions. Triplicate mean fitted to the Weibull kinetic model.

A quantitative analysis of the release of DOX from DPPC/DSPC/CHEMS-based MLs, under mimetic therapeutic and physiological conditions, was carried out. These assays indicate the ability of MLs to act as an effective controlled drug delivery system under specific conditions. Moreover, it allows an understanding of the influence of lipid formulations on the release profile, and the partial prediction of the in vivo behavior and the ability to sustain therapeutic levels over an extended period. Thus, the ability of nanosystems to respond in a controlled manner was evaluated in four combinations of therapeutically (42 °C and pH 5.5) and physiologically (37 °C and pH 7.4) relevant stimuli. Figure 10 summarizes DOX release kinetics.

The results revealed a temperature-dependent drug release rate, with a notable increase in release at 42 °C (red lines), simulating hyperthermia conditions, compared to the release observed at physiological temperature (black lines). Previous studies have demonstrated that the lipid combination investigated in this work has a transition temperature of 39.7 ± 0.1 °C [60]. DOX release at 42 °C was found to be highest at pH 5.5, reaching (23 ± 1)% at 26 h, whereas at pH 7.4 this value was (16 ± 3)%. These values result from the increased hydrophilicity of DOX in acidic environments, already demonstrated in previous works [58]. In contrast, experiments conducted at 37 °C did not show significant differences between releases at pH 5.5 and 7.4, with both exhibiting similar values of (10.4 ± 0.3)% and (10 ± 1)% of release at 26 h, respectively. Moreover, the experiments at 37 °C demonstrated a desirable delayed release of DOX. These results highlight a synergistic effect between acidic pH and hyperthermia, making this combination ideal for the application proposed in this work. This temperature-dependent control allows for the effective shielding of drugs under non-therapeutic conditions, reducing off-target effects and enhancing therapeutic concentrations specifically at the target sites.

**Figure 11:**
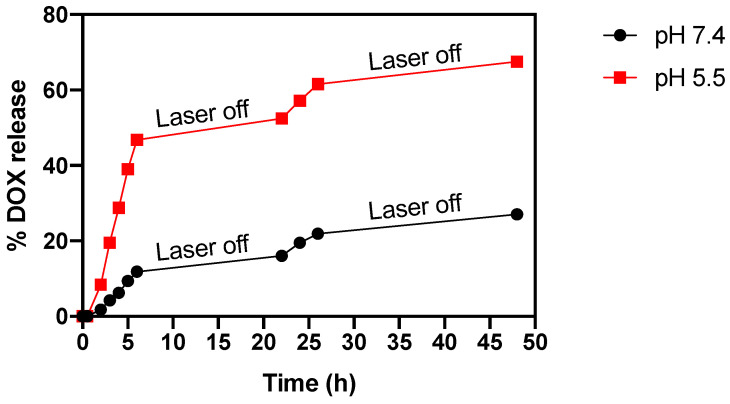
Release profile of DOX from DPPC/DSPC/CHEMS-based MLs at room temperature under different pH conditions by repeated laser irradiation.

To investigate the combined effect of photothermal hyperthermia on the drug release behavior of MLs, a series of tests were conducted under repetitive laser irradiation, both at room temperature and at different pH levels (pH 7.4 and 5.5). The findings are displayed in Figure 11, revealing a compelling synergistic effect of temperature, especially in conjunction with pH 5.5.

The results demonstrated that laser irradiation destabilized the MLs’ membrane, leading to significantly greater drug release compared to tests simulating hyperthermia at 42 °C. For instance, at 6 h, the sample subjected to 42 °C and pH 5.5 exhibited (17 ± 2)% DOX release, while under laser irradiation, the release escalated to (46.7 ± 0.6)%. The MLs’ capacity to retain DOX during periods without laser irradiation was also assessed. Only a residual release of DOX was observed during this laser-off period between 6 and 22 h, indicating the effective drug containment within the MLs. To further validate the MLs’ ability to retain DOX, the test was extended to 48 h with the laser off. Once again, under both conditions, the release of DOX remained minimal. At the end of the assay, at pH 5.5, (67.5 ± 0.5)% of DOX was released, whereas at pH 7.4, only (27.0 ± 0.1)% was released. These results evidence that the MLs synthesized in this study not only effectively control the release of DOX under NIR-laser stimulation, but also potentiate this release in the presence of a tumor microenvironment, characterized by a more acidic pH. In contrast, the MLs maintain a sustained and reduced release profile in physiological environments with pH 7.4.

## 4. Conclusions

The comprehensive analysis presented in this work provides compelling evidence for the successful functionalization of shape-anisotropic Ca_0.25_Mg_0.75_Fe_2_O_4_ nanoparticles with dimercaptosuccinic acid (NPs@DMSA), resulting in the formation of hydrophilic nanostructured nanoparticles. Absorption spectroscopy demonstrated the DMSA functionalization by revealing increased absorption and light scattering for NPs@DMSA compared to the non-functionalized nanoparticles. The photothermal hyperthermia assays conducted under laser irradiation at 808 nm for 30 min revealed significant temperature variations: 20.4 °C, 11.4 °C, and 7.3 °C for NPs@DMSA concentrations of 4 mg/mL, 2 mg/mL, and 1 mg/mL, respectively. These results highlight the photothermal properties of NPs@DMSA, as demonstrated by the high specific absorption rate (SAR) values ranging from 3338.34 W/g to 4897.62 W/g. Furthermore, the superparamagnetic behavior of NPs@DMSA was observed, although their saturation magnetization was approximately nine times lower compared to the non-functionalized nanoparticles.

A novel synthesis method was developed to couple the prepared NPs@DMSA onto the liposomal lipid bilayer’s surface. Based on the group’s previous results, the lipid formulations of DPPC/DSPC/CHEMS and DPPC/DSPC/CHEMS/DSPE-PEG were chosen as the lipid components for the preparation of the MLs. To confirm the coupling of NPs@DMSA to the lipid surface of the MLs, ζ-potential-pH profile assays were conducted on NPs@DMSA, liposomes, and the prepared MLs. The observed MLs’ mixed profile of liposomes and magnetic nanoparticles confirmed the formation of hybrid complexes.

The morphology and size of the MLs were further confirmed using scanning electron microscopy (SEM), which revealed the presence of rounded-shaped MLs with an irregular surface and high contrast. The DPPC/DSPC/CHEMS-based MLs exhibited a size of 153 ± 8 nm, while the DPPC/DSPC/CHEMS/DSPE-PEG-based MLs had a size of 136 ± 2 nm. These sizes are well suited for various biomedical applications. Additionally, the DPPC/DSPC/CHEMS-based MLs demonstrated a higher encapsulation efficiency of NPs@DMSA onto the lipid surface (EE_NPs@DMSA_ = 61 ± 3%) compared to the stealth MLs (EE_NPs@DMSA_ = 17 ± 1%). Based on these findings, the DPPC/DSPC/CHEMS-based MLs were selected as the optimal nanosystems for further investigations. DOX release kinetics assays were conducted under physiological and therapeutically relevant conditions. The results indicated a higher release rate at 42 °C, suggesting the potential of the MLs for controlled drug release in response to temperature changes. Moreover, the introduction of laser-induced photothermal hyperthermia at 808 nm (1 W/cm^2^) exhibited a remarkable synergistic effect on the DOX release profile, exceeding the release observed with a mimetic effect of hyperthermia (42 °C) at both pH 5.5 and 7.4. The findings suggest that the produced MLs can release DOX selectively in response to NIR-laser stimulation, making them suitable candidates for targeted drug delivery in cancer therapy. Furthermore, the ability to explore the tumor’s acidic milieu to increase drug release while preserving control under normal physiological conditions has enormous potential for improving therapeutic outcomes and lowering systemic adverse effects.

Overall, the results indicate that using NPs@DMSA coupled with thermosensitive lipid bilayers is a promising approach for dual cancer therapy, combining chemotherapy and photothermal hyperthermia in oncology treatments.

## Figures and Tables

**Table 1:** Lipid composition and molar ratio of lipids used in the synthesis of nanosystems.

Lipid Composition	Ratio	Type of Lipid Nanosystem
DPPC	1	Thermosensitive
DPPC/DSPC/CHEMS	7:2:1	Medium/long circulation, fusogenic, thermosensitive, and pH-sensitive
DPPC/DSPC/CHEMS/DSPE-PEG	60:20:15:5	Long circulation, fusogenic, thermosensitive, and pH-sensitive

**Table 2:** Saturation magnetization (M_s_), Coercive field (H_C_), remnant magnetization (M_r_), and M_r_/M_s_ ratio for nanoparticles functionalized and non-functionalized with DMSA.

Sample	M_s_ (emu/g)	H_c_ (Oe)	M_r_ (emu/g)	M_r_/M_s_
NPs	50.13	1.85	0.07	0.001
NPs@DMSA	5.66	33.92	0.28	0.049

**Table 3:** Hydrodynamic size, polydispersity index, and encapsulation efficiency of DOX and magnetic nanoparticles in liposomes and magnetoliposomes of different lipid compositions.

Nanosystem	Lipid Compositions	D_H_ (nm)	PDI	EE_DOX_ (%)	EE_NPs@DMSA_ (%)
Liposomes	DPPC	118 ± 5	0.26 ± 0.01	98.3 ± 0.8	---
MLs	DPPC/DSPC/CHEMS	153 ± 8	0.22 ± 0.03	96 ± 2	61 ± 3
MLs	DPPC/DSPC/CHEMS/DSPE-PEG	136 ± 2	0.24 ± 0.01	98.0 ± 0.6	17 ± 1

**Table 4:** The dissociation constant ($k_{d}$), binding constant ($k_{b}$ = 1/$k_{d}$), and the number of binding sites (n) of DOX-loaded liposomes and MLs.

	$k_{d}$ (M)	$k_{b}$ (M^−1^)	n	**R^2^**
Free DOX	8.24 × 10^−7^	1.21 × 10^6^	1.80	0.96
DPPC-based liposomes	4.41 × 10^−7^	2.27 × 10^6^	0.90	0.99
DPPC/DSPC/CHEMS-based MLs	2.44 × 10^−7^	4.10 × 10^6^	1.10	0.93
DPPC/DSPC/CHEMS/DSPE-PEG-based MLs	5.87 × 10^−6^	1.70 × 10^5^	1.16	0.97
NPs@DMSA	2.79 × 10^−7^	3.58 × 10^6^	0.62	0.13

## Data Availability

Not applicable.
